# Fast, automated measurement of nematode swimming (thrashing) without morphometry

**DOI:** 10.1186/1471-2202-10-84

**Published:** 2009-07-20

**Authors:** Steven D Buckingham, David B Sattelle

**Affiliations:** 1 MRC Functional Genomics Unit, Department of Physiology, Anatomy and Genetics, University of Oxford, South Parks Road, Oxford, OX1 3QX, UK

## Abstract

**Background:**

The "thrashing assay", in which nematodes are placed in liquid and the frequency of lateral swimming ("thrashing") movements estimated, is a well-established method for measuring motility in the genetic model organism *Caenorhabditis elegans *as well as in parasitic nematodes. It is used as an index of the effects of drugs, chemicals or mutations on motility and has proved useful in identifying mutants affecting behaviour. However, the method is laborious, subject to experimenter error, and therefore does not permit high-throughput applications. Existing automation methods usually involve analysis of worm shape, but this is computationally demanding and error-prone. Here we present a novel, robust and rapid method of automatically counting the thrashing frequency of worms that avoids morphometry but nonetheless gives a direct measure of thrashing frequency. Our method uses principal components analysis to remove the background, followed by computation of a covariance matrix of the remaining image frames from which the interval between statistically-similar frames is estimated.

**Results:**

We tested the performance of our covariance method in measuring thrashing rates of worms using mutations that affect motility and found that it accurately substituted for laborious, manual measurements over a wide range of thrashing rates. The algorithm used also enabled us to determine a dose-dependent inhibition of thrashing frequency by the anthelmintic drug, levamisole, illustrating the suitability of the system for assaying the effects of drugs and chemicals on motility. Furthermore, the algorithm successfully measured the actions of levamisole on a parasitic nematode, *Haemonchus contortus*, which undergoes complex contorted shapes whilst swimming, without alterations in the code or of any parameters, indicating that it is applicable to different nematode species, including parasitic nematodes. Our method is capable of analyzing a 30 s movie in less than 30 s and can therefore be deployed in rapid screens.

**Conclusion:**

We demonstrate that a covariance-based method yields a fast, reliable, automated measurement of *C. elegans *motility which can replace the far more time-consuming, manual method. The absence of a morphometry step means that the method can be applied to any nematode that swims in liquid and, together with its speed, this simplicity lends itself to deployment in large-scale chemical and genetic screens.

## Background

The nematode, *Caenorhabditis elegans*, has been used extensively for tackling fundamental questions in biology, including the modeling of aspects of human disease, drug screening and development [1-5]. It was the first complex animal genome to be sequenced [6]. Its high fecundity, short life cycle and the availability of many mutants facilitate the exploration of gene function, while its complete cell lineage and transparency are useful in studies of development. The discovery of the RNA interference method for selective gene knockdown by Fire and Mello [7] and the ease of delivery, via *Escherichia coli *on which the worms feed, of specifically-targeted double-stranded RNA has paved the way for genome-wide RNAi screens [8]. However, despite the facility with which large-scale genetic and chemical screens can be applied to *C. elegans*, the lack of automation of phentoyping has limited its use in highly automated screening applications.

To fully harness the potential of the growing number of *C. elegans *disease models for discovering new drugs, it is therefore necessary to develop automated methods of assaying locomotor phenotypes. For example, screening for new anthelmintics using *C. elegans *or parasitic worms could be accelerated if automated phenotyping could be deployed in screening chemical libraries. Parasitic nematodes present a major challenge to both human and animal health [9,10] so automation could also assist in accelerating screening for the discovery of new antiparasitic drugs. In addition, automation would enhance the utility of *C. elegans *models mimicking aspects of human nervous system and neuromuscular diseases.

Assays of *C. elegans *motility frequently deploy the "thrashing assay". This is performed by placing worms in liquid medium and counting the number of lateral swimming movements (thrashes min^-1^), thereby quantifying an important aspect of locomotion. The effects on locomotion of genetic manipulations and/or drugs can thus be studied with little training, yielding a numerical output that is easy to relate to behaviour. The manual assay, however, suffers two major disadvantages. First, it is very time consuming. Each measurement takes at least 30 s to perform, making it unsuitable for high-throughput assays. In addition, since it requires a human operator, it can be prone to errors in counting, especially at high thrashing rates (over 4 s^-1 ^in a healthy, wild-type worm) with the added hazard of investigator repetitive strain. These difficulties, when set against the tremendous potential for drug/chemical discovery if *C. elegans *could be used for automated chemical and genetic screens, point to the urgent need for a simple, automated system of counting thrashes.

Current approaches to automating the thrashing assay follow two general strategies [11]. The first is to emulate the human measurement using computer vision. Typically, the worm is distinguished from the background and its shape estimated. The body angles through which the worms pass are then determined, from which the frequency of thrashing can be measured. This approach provides a readout that can be compared directly with manually-derived values. Only two such analyses, designed specifically for the thrashing assay, have been described. In both cases, several parameters in addition to thrashing rate have been measured [12,13] but the software for these analyses has not been published. Several tools for the analysis of nematode locomotion on agar, rather than in fluid, have been made available which could be applied to analyzing thrashing worms [14-20]. Although providing extra information additional to thrashing rate, this strategy is subject to several difficulties. For example, its accuracy depends on precise morphometry, which in turn depends upon accurately distinguishing the worm from its background (referred to as "segmentation" in the context of computer vision). These processes are usually highly sensitive to recording conditions and can fail under uneven illumination. Even after accurate morphometric data have been obtained, estimating thrashing frequency from the data presents further problems. The most obvious method, that of counting the peaks in a graph of angle against time, can be confounded by the presence of random fluctuations in the measured angle, and setting the threshold to distinguish real peaks from noise in different experimental trials can be difficult. The alternative method of using Fourier analysis to derive the strongest component in the frequency domain can fail at low frequencies, where the signal components are hard to distinguish from the DC signal.

A second approach replaces the manual assay with an alternative, less direct method of estimating thrashing frequency. For instance, Simonetta and Golombek [21] measured thrashing of several worms simultaneously by recording the interruption of an infrared beam. Although such indirect measures do have potential for automation and high-throughput screening, the output of such an assay is not easily related to manual methods, making the results difficult to validate and to interpret biologically.

## Implementation

Here we present a method of automatically measuring thrashes per minute, but without using morphometry or even segmentation of the worm from the background. In our approach, the covariance matrix of film frames of thrashing worms is used to measure the time interval between frames that are statistically similar.

**Figure 1:**
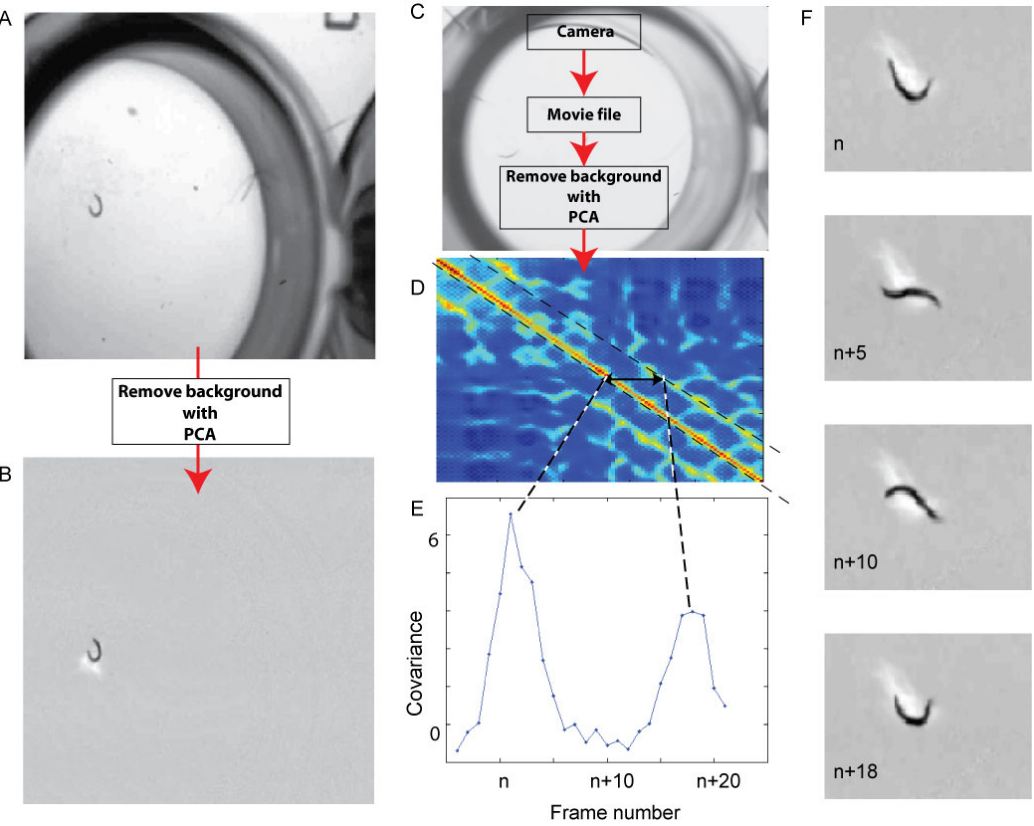
**Covariance analysis of worm swimming facilitates automated phenotyping.** (A) A typical image from a movie of a worm thrashing in a well of a 96-well plate. (B) The same image, after removal of the first Principal Component to eliminate most of the image background. (C) A summary of the method used to measure worm swimming rates using covariance. After acquisition with a digital camera attached to a stereomicroscope, movies are stored for subsequent analysis. (D) After subtracting the background using Principal Components Analysis, a covariance matrix is computed. (E) The number of frames separating two peaks in covariance is equal to the interval over which the worm has undergone a complete cycle of conformations, and is therefore twice the interval between thrashes. (F) A sequence of background-subtracted images corresponding to the points in (D) and (E) covering a single thrash cycle.

Our algorithm follows three steps (Figure 1). First, the presence of the image background is reduced using Principal Components Analysis[22,23]. To accomplish this, a movie of the worm, consisting of *d m *× *n *pixel images, is loaded into a *m *× *n *× *d *3-dimensional array, which is then transformed into a 2 dimensional matrix, with each frame being contained in a single column. The first Principal Component of this matrix accounts for more than 99% of the variance. Examination of the image data contained in this component showed that it represents, as would be expected, the background of the images, while the remaining components are attributable to the worm conformation (Figure 1A, B). Thus, subtracting the first Principal Component from the 2-dimensional matrix reduces the presence of background, after which the movie can be reconstructed.

After reduction of the background, the next step is to calculate the covariance matrix. The covariance matrix of the films after removal of the first Principal Component shows a diagonal band of high values, representing the variance of each frame, with similar, parallel bands of lower peak heights (Figure 1). These parallel bands represent frames with high covariance separated by frames with low covariance. The number of frames separating the peaks correspond to the interval between similar images and hence to the interval between similar worm conformations.

The third step in our algorithm consists of deriving the thrashing frequency from the covariance matrix. We compared two methods of automating the measurement of the interval between these peaks. First, we tried converting the covariance matrix into the frequency domain using the Discrete Fourier Transform. This gave accurate measurements of the peak interval at high thrashing rates, but at low rates the peaks corresponding to thrashes became hard to distinguish without human intervention. We therefore adopted the *peakdet.m *routine generously released into the public domain by Mr. Eli Billauer  in which frames separating peaks greater than 50% of the total range of data are counted for each point along the diagonal of the covariance matrix. The thrashing frequency is taken as the product of the median of these values and twice the film acquisition frequency. The median is preferred over the mean, as the former is less sensitive to the effect of outliers. This median is multiplied by twice the acquisition frequency because the strongest covariance is between worm conformations at the same phase of swimming, which represents the movement between two thrashes, as one thrash is counted as a single side-to-side movement. This method has proved robust at all thrashing rates up to about 300 s^-1^, this upper limit being presumably determined by the film acquisition frequency.

## Data acquisition

*C. elegans *were raised at 21°C under standard laboratory conditions on agar plates seeded with a lawn of *E. coli *(OP50) on which they feed. The following *C. elegans *strains were used: N2 wild-type (Bristol variety), as well as nicotinic acetylcholine receptor mutants *unc-63(x26), unc-63(x37), lev-8, acr-16, lev-1, unc-29 *and *unc-38*. All strains were handled according to standard procedures . *Haemonchus contortus *L3 larvae were kindly supplied by Dr. Adrian Wolstenholme of University of Bath.

Worms were synchronously grown to early adult stage and placed in individual wells of a 96-well microtiter plate containing 50 μl M9 with or without drug. After a 10 min exposure period to M9, thrashes were counted at 21°C for 30 s. A single thrash was defined as a complete change in the direction of bending at the mid body. Worms were either counted and filmed at the same time, or filmed and subsequently counted from the movies. Manual counting was performed independently by two trained experimenters. For automated analysis, each experiment was processed as a batch. In accordance with accepted practice, during manual thrash counts the data for a particular worm was dropped if the worm remained still for > 10 s, or if the worm was visibly damaged. In automated assays, all worms were included. Worms were filmed using an XLI 2 Mpixel camera attached to a Nikon SMZ 1000 dissecting microscope and the 640 × 320 pixel images acquired at 10 frames s^-1 ^using XLI imaging software. Before filming, the image was magnified and positioned so that the circular well of the microtitre plate occupied the field of view. Movies were stored in the Microsoft "wmv" format and subsequently analysed using the Matlab software package running on a desktop PC running under Windows XP. To speed computation, images were reduced to 20% of their resolution.

## Results

**Figure 2:**
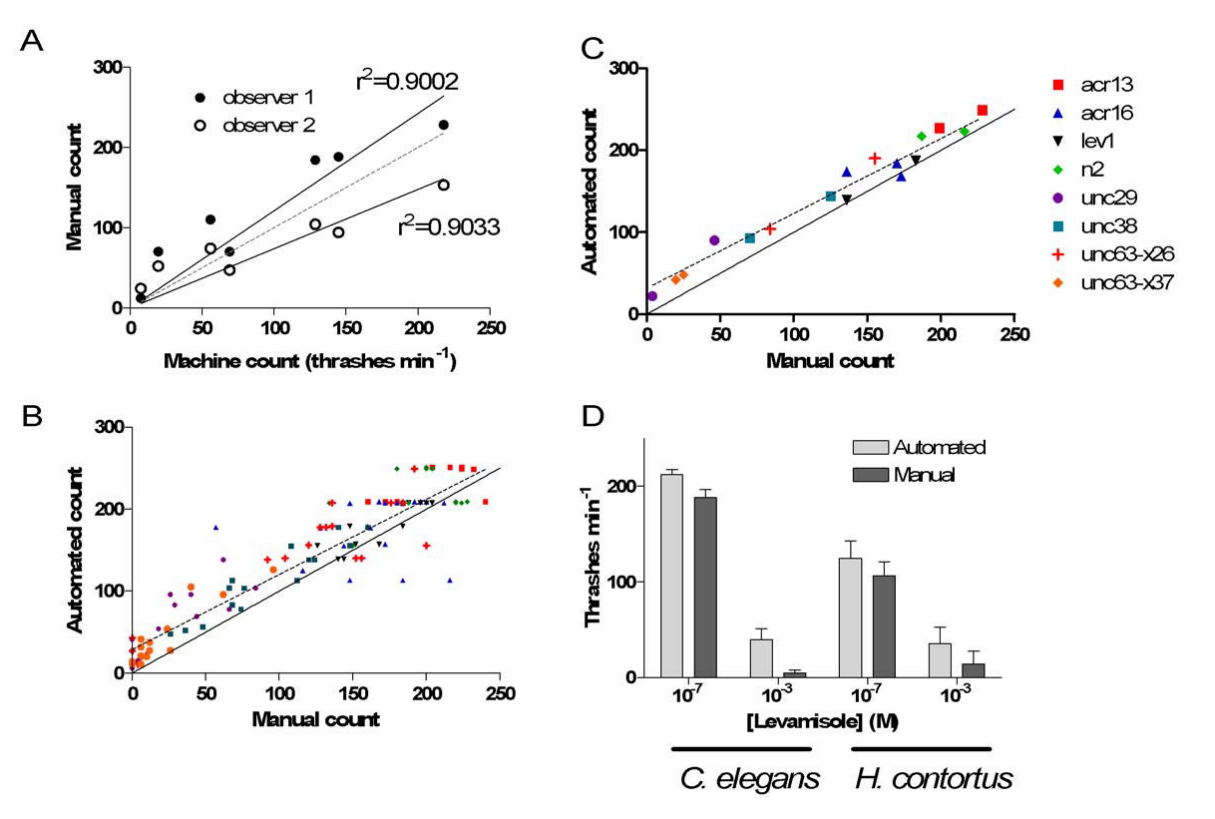
**Comparison of automated thrashing assay with manual measurements**. (A) The performance of the machine method on *C. elegans *is compared with that of two human observers. The variance in the automated system's performance can therefore be compared with the difference in performance between two trained observers. Each point represents the mean of counts for 8 worms, in accordance with the customary thrashing assay. (B) Comparison of results for individual *C. elegans *with mutations in nicotinic acetylcholine receptors with ranges of motilities. (C) Although the machine frequently underestimates the counts because of outliers, the effect of these outliers in each batch of 8 worms is reduced when the medians for each method are plotted. (D) A comparison of automated and manual thrashing assays on *C. elegans *or the parasitic nematode, *Haemonchus contortus*, in the presence of two concentrations of levamisole. Despite the challenges presented by *H. contortus*, the automated assay produces similar results to manual measurements.

To assess the performance of the system, we carried out a series of thrashing assays on wild-type (N2) worms along with several mutants of the levamisole receptor (*unc-29, unc-38, lev1, unc-63 and lev-8*), which have a range of effects on motility. We used the *x26 *allele of the *unc-63 *mutant because the mutation which opens up the dicysteine loop mimics a mutation producing the same effect in patients with one form of human congenital myasthenia [24]. Plotting the individual machine counts for all these receptor mutants against manual counts revealed a linear relationship between these measurements with a correlation coefficient greater than 0.9 (Figure 2A), suggesting that the machine performance was comparable to manual measurement. When individual scores obtained using machine and manual methods are compared (Figure 2B), it can be seen that the automated system tends to slightly overestimate the thrashing frequency, but if the median of each trial is taken (the mean is used in manual assays), the effect of these outliers is limited and the system compares very favourably with manual counts (Figure 2C).

To test the usefulness of our method in analyzing the effects of drugs on motility, we performed a thrashing analysis of the dose-dependence of the actions of the anthelmintic drug, levamisole, on wild-type (N2) worms, comparing counts obtained by a human observer with those obtained using our automated covariance method. Both approaches revealed a similar dose-dependent inhibition of thrashing frequency (Figure 2D).

Because the method makes no assumptions about the shape of the worms, it is adaptable for use with other nematode species. We repeated our measurement of levamisole effects using 3rd instar larvae of the parasitic nematode, *Haemonchus contortus*. The parasite's swimming behaviour at low rates often involves the animal completely coiling up on itself, which would represent an insurmountable difficulty for most, if not all, machine-vision approaches. The algorithm revealed a dose-dependent action of levamisole closely resembling that measured manually (figure 2D).

## Discussion

Our method of automating thrashing assays has a number of advantages over traditional machine-vision approaches. Avoiding the use of morphometry speeds computation. As implemented in our Matlab script, the method is capable of analyzing a previously recorded 30 s movie off-line in less than 30 s, and can therefore be applied to directly-acquired images without the need to store movies. This is an important consideration in developing high-throughput screens, as moving images place high demands on computer storage space. In addition, errors introduced by failures in accurately distinguishing the worm from its background or in abstracting morphometric parameters are avoided. Finally, because it makes no assumptions about the shape of the worms, it is robustly adaptable for use with other species, as shown here by our study on the parasitic worm, *H. contortus*.

We have implemented this novel method using films of single worms in individual wells of a 96-well plate so that its output with manual assays can be directly compared. Deployed in this way, it can only be applied to one worm at a time, as the presence of more than one worm in the field of view (unless it is significantly smaller) will make it difficult to derive an accurate measurement of thrashing frequency from the covariance matrix. However, adaptations of the same principle could be used to increase the number of worms that can be measured simultaneously. For example, an array of cameras could be used to acquire data from several wells simultaneously. Alternatively, if the worms can somehow be spatially restricted, for example, in plate wells, a large number of worms could be monitored simultaneously with a single camera if the images are split to cover each worm separately.

## Conclusion

Here we present a method for automating an established swimming ("thrashing") assay widely used for measuring locomotor phenotypes of the model genetic organism, *Caenorhabditis elegans*. Measurements of the frequency of thrashing and their reliability compare well with manual counting over the entire range of thrashing rates likely to be encountered in studying *C. elegans*. Thus, our method of automating worm thrashing assays using covariance is robust, reliable and fast. Deployed in a fully automated system incorporating liquid-handling systems to deliver worms in suspension to microplates and computer-controlled positioning of the plate, this method should facilitate large-scale chemical and genetic screens for effects on motility.

## Availability and requirements

*Project name*: Automated thrashing assay using covariance

*Operating system*: Platform independent

*Programming language*: Matlab script

*Other requirements*: Matlab, peakdet.m

*Licence*: GNU GPL

*Any restrictions to use by non-acedemics*: licence needed

## Authors' contributions

DBS guided the development of the study and its application to screening, SDB conceived of, developed and tested the software.
